# Supplementary material for: Exploring current and potential roles of informal healthcare providers in tuberculosis care in West Bengal, India: A qualitative content analysis
Source: PLOS Glob Public Health. 2025 Nov 5;5(11):e0004085. doi: 10.1371/journal.pgph.0004085 (PMC12588489; doi:10.1371/journal.pgph.0004085)
Supplement: S1 Text — (PDF) [file pgph.0004085.s001.pdf]

Table 1: Roles of IPs in TB care (classified based on TB care functions)

Types of care		IPs TB care roles (by functions) *	Definitions		Refinements of roles based on the qualitative study findings **	Definitions
**Prevention**	1	Health promotion and education	Services include (but are not limited to) awareness-raising and social mobilization activities.	1	Health promotion and education	Activities related to promoting information on TB in the community or providing health education to any patient visiting IPs clinic before being diagnosed as having TB.
	2	Immunization	Activities related to BCG Vaccination	2	Immunization	Activities related to BCG Vaccination
	3	Latent TB infection screening	Activities related to screening of latent tuberculosis	3	Latent TB infection screening	Activities related to screening of latent tuberculosis
	4	Latent TB infection prescription	Activities related to prescription of preventative treatment	4	Latent TB infection prescription	Activities related to prescription of preventative treatment
	5	Latent TB infection administration	Activities related to administration of preventative treatment	5	Latent TB infection administration	Activities related to administration of preventative treatment
**Detection and diagnosis**	6	Active case finding	Activities related to active finding of TB cases in any setting	6	Active case finding	Activities related to active finding of TB cases in any setting
	7	Passive case finding and referral	Activities related to screening and referral of TB cases among patients who visit IPs clinic	7	Passive case finding and referral	Activities related to screening and referral of TB cases among patients who visit IPs clinic
				8	Accompany suspected TB cases to a health facility	Activities related to accompanying a suspected case of TB to a health facility
	8	Clinical evaluation-TB	Activities related to clinical evaluation of TB among suspected cases	9	Clinical evaluation-TB	Activities related to clinical evaluation of TB among suspected cases
	9	Laboratory examination and/or X-ray	Activities related to laboratory examination of TB specimens and X-ray examination	10	Collection and transportation of sputum samples	Activities related to collection and transportation of sputum samples to designated facilities
				11	Contact tracing	Activities related to screening of contacts who were exposed to a case of confirmed TB
**Treatment and support**	10	Treatment initiation	Activities related to prescription of TB drugs to confirmed patients	12	Treatment initiation	Activities related to prescription of TB drugs to confirmed patients
	11	Treatment supporter	Activities related to supporting TB treatment	13	Treatment supporter	Activities related to supporting TB treatment
	12	Monitoring treatment progress and response	Activities related to periodic clinical evaluation and lab monitoring	14	Monitoring treatment progress and response	Activities related to periodic clinical evaluation and lab monitoring
	13	Prevention and detection of adverse events and comorbidities	Activities related to monitoring of adverse drug reactions	15	Prevention and detection of adverse events and comorbidities	Activities related to monitoring of adverse drug reactions
	14	Diagnosis and treatment of adverse events and comorbidities	Activities related to treatment of adverse drug reactions	16	Diagnosis and treatment of adverse events and comorbidities	Activities related to treatment of adverse drug reactions

	15	Treatment lab monitoring	Activities related to conduct of laboratory examinations during treatment	17	Treatment lab monitoring	Activities related to conduct of laboratory examinations during treatment
	16	Counselling and psychological support	Activities related to providing counselling and psychological support during treatment	18	Counselling – During TB treatment	Activities related to providing counselling and psychological support during treatment
	17	Social support	Activities related to social support for a TB patient	19	Social support	Activities related to social support for a TB patient

*IPs' roles identified in the scoping review

**IPs' roles refined based on the qualitative study findings
