# Supplementary material for: Exploring current and potential roles of informal healthcare providers in tuberculosis care in West Bengal, India: A qualitative content analysis
Source: PLOS Glob Public Health. 2025 Nov 5;5(11):e0004085. doi: 10.1371/journal.pgph.0004085 (PMC12588489; doi:10.1371/journal.pgph.0004085)
Supplement: S1 Checklist — (DOCX) [file pgph.0004085.s004.docx]

Inclusivity in global research

PLOS’ policy on inclusivity in global research aims to improve transparency in the reporting of research performed outside of researchers’ own country or community and ensures that PLOS publications reporting global research adhere to high standards for research ethics and authorship. Authors of relevant research articles may be asked to complete the questionnaire below, which outlines ethical, cultural, and scientific considerations specific to inclusivity in global research. This questionnaire may be requested when researchers have travelled to a different country to conduct research, if research uses samples collected in another country, research with Indigenous populations or their lands, or if research is on cultural artefacts. Researchers travelling to another country solely to use laboratory equipment will not normally be required to complete the questionnaire. However, the questionnaire can be requested at the journal’s discretion for any submission – if you have been requested to complete this questionnaire by the PLOS journal you submitted to, please do so.

Please complete the questionnaire below and include this as a Supporting Information file with your manuscript. Note that if your paper is accepted for publication, this checklist will be published with your article in the supporting information files. Please ensure that you reference the checklist in the main body of your manuscript. We suggest adding a subsection ‘Inclusivity in global research’ to your Methods section and adding the following sentence: “Additional information regarding the ethical, cultural, and scientific considerations specific to inclusivity in global research is included in the Supporting Information (SX Checklist)”

The questions have been designed to be applicable to a wide range of study types, and there are subsections for both human subjects research and non-human subjects research. If any of the questions are not relevant to your research please mark them as “N/A” as appropriate.

**Ethical considerations, permits and authorship**

*This section is applicable to all research types.*

Provide details as to who granted permissions and/or consent for the study to take place in the Methods section of your manuscript. This should include the names of **all** ethics boards, governmental organizations, community leaders or other bodies that provided approval for the study. If individuals provided approval refer to these people by their role or title but do not list their name(s).

Ethical approval was obtained from the University of New South Wales Human Research Ethics Committee (HC210258) and the LFWB Human Research Ethics Committee (IILDS/IEC/001). These approvals covered all aspects of the study, including remote interviews and verbal informed consent, as described in the Methods [Page number: To be added].

If there were any deviations from the study protocol after approval was obtained please provide details of these changes in the Methods section of your manuscript.

No deviations from the approved protocol were made.

Did this study involve local collaborators that are residents of the country where the research was conducted or members of the community studied? If you do not have any authors from said communities, please provide an explanation for this below.

Yes, the study involved local collaborators residing in West Bengal, India. Two co-authors are affiliated with implementing organizations (Liver Foundation) based in the study setting, and the authorship order is based on the contribution to the project. These collaborators were actively involved in study design, implementation, data collection, and manuscript review.

Everyone listed as an author should meet PLOS’ criteria for authorship and all individuals who meet these criteria should be included in the author byline, rather than the acknowledgements. Authorship criteria is based on the International Committee of Medical Journal Editors (ICMJE) Uniform Requirements for Manuscripts Submitted to Biomedical Journals - for further information please see here: <https://journals.plos.org/plosone/s/authorship>.

**Human subjects research (e.g. health research, medical research, cross-cultural psychology)**

Did you obtain written informed consent from a representative of the local community or region before the research took place? How did you establish who speaks for the community? Details of written informed consent obtained from study participants should be reported separately in the Methods section of your manuscript.

The study was discussed and approved by the state and district TB offices, who are responsible for overseeing the TB program at the respective levels. All research activities described in the Methods were approved by local ethics boards (LFWB Human Research Ethics Committee). The study involved individual interviews with providers and NTEP-affiliated personnel. Each participant gave informed verbal consent, which was audio-recorded and stored separately from the main data to maintain confidentiality.

How did members of the local community provide input on the aims of the research investigation, its methodology, and its anticipated outcome(s)?

The Liver Foundation, our local partner, has a long-standing relationship with the State and District TB offices, with several ongoing collaborative projects at the time of the study. Personnel from the State and District TB offices reviewed the study protocol and tools, and their input was gathered and incorporated into the final instruments. The study aims and methodology were developed in collaboration with the Liver Foundation to ensure contextual relevance and alignment with existing TB programmatic priorities.

When engaging with the local community, how did you ensure that the informed consent documents and other materials could be understood by local stakeholders?

All interview guides and consent materials were translated into Bengali, and interviews were conducted by trained research staff fluent in local languages and familiar with the sociocultural context.

Will the findings of the research be made available in an understandable format to stakeholders in the community where the study was conducted (e.g. via a presentation, summary report, copies of publications, etc.)? Please provide details of how this will be achieved.

As this study was part of a larger multi-method research project, all publications resulting from the project have been routinely shared with the State and District TB offices. A co-author (PSM), affiliated with the Liver Foundation, personally ensured that each publication was delivered to the relevant officials, either via email or in person, based on their stated preference. We intend to follow the same practice for this manuscript and will share the published article with the concerned stakeholders to support local dissemination and use.

**Non-human subjects research using specimens/ animals collected as part of the study, or those housed in archival collections. Examples include archaeology, paleontology, botany and zoology.**

Did the permission you obtained from a local authority to perform the study include an agreement on access to outputs and benefit sharing? This may include procedures to enable fair distribution of the benefits and resources arising from the research performed. Please include any details of Prior Informed Consent and Benefit Sharing Agreements obtained. These may be required by field-specific regulations, for example the Convention on Biological Diversity (CBD) and the associated Nagoya Protocol.

Not applicable

If the material used in your study was imported, please A) provide the year it was imported and B) indicate whether permits were obtained to import/export the materials used, C) provide details of any permits obtained. If this information is not available, please indicate this.

Not applicable

If you used archival specimens, please state how the material used in your study was acquired by the institute it is held in and provide details of any permits obtained for the original excavations/ sample collection. If this information is not available, please indicate this.

Not applicable

How was the potential cultural significance of the materials collected in your study to local communities considered in your research design? Were Indigenous peoples and/or local researchers and institutions involved with archaeological excavations / collection of specimens? If so, please provide a description of their involvement.

Not applicable

If your manuscript includes photographs of human remains please indicate whether authors obtained permission from descendants or affiliated cultural communities to do so.

Not applicable
