# Supplementary material for: Exploring current and potential roles of informal healthcare providers in tuberculosis care in West Bengal, India: A qualitative content analysis
Source: PLOS Glob Public Health. 2025 Nov 5;5(11):e0004085. doi: 10.1371/journal.pgph.0004085 (PMC12588489; doi:10.1371/journal.pgph.0004085)
Supplement: Attachment — Submitted filename: REBUTT~1.DOC [file pgph.0004085.s006.DOC]

29 April 2025

The Editor,

PLOS Global Public Health,

Dear Editor,

**Re: Exploring current and potential roles of informal healthcare providers in tuberculosis care in West Bengal, India: a qualitative content analysis**

We thank you and the external reviewers for the valuable comments that have allowed us to strengthen our manuscript. We have carefully reviewed the manuscript, and below, we provide a detailed response to reviewers' comments. We have also provided a tracked change version of the manuscript, highlighting the changes made to the originally submitted version.

**Journal Requirements:**

1. In the ethics statement in the Methods, you have specified that verbal consent was obtained. Please provide additional details regarding how this consent was documented and witnessed, and state whether this was approved by the IRB

**Response:** Thank you for this important comment. We have revised the ethics statement in the Methods section as follows:

*Ethical approval for this study was obtained from the UNSW Human Research Ethics Committee (HC210258) and the LFWB Human Research Ethics Committee (IILDS/IEC/001). Informed verbal consent was obtained from all participants, in accordance with the procedures approved by both ethics’ committees. Before data collection, interviewers verbally administered a standardized consent script and provided participants with the opportunity to ask questions. Upon confirming their understanding, participants’ verbal consent was audio-recorded. To maintain confidentiality, consent recordings were securely stored and kept separate from all other study data.*

**Please refer to line 246 on page 9 of the revised manuscript (clean version).**

2. Please include a complete copy of PLOS’ questionnaire on inclusivity in global research in your revised manuscript. Our policy for research in this area aims to improve transparency in the reporting of research performed outside of researchers’ own country or community. Please upload a completed version of your questionnaire as Supporting Information when you resubmit your manuscript.

**Response:** Thank you for this guidance. As requested, we have completed the PLOS questionnaire on inclusivity in global research and included it as a Supporting Information file in our revised submission.

**Reviewer 1:**

3. The work presented here explores current and potential roles of a subset of informal providers – Untrained Allopathic Practitioners (UAPs) – in TB care in West Bengal in India. Overall, the research employed sound methodology by drawing on perspectives of both informal and formal providers to identify these roles. In particular, it is thoughtful to include formal providers because UAPs sometimes share a close relationship with the formal providers (particularly ASHA in this case). From a methodological perspective, use of the content analysis methodology outlined by Elo and Kygnas, drawing on previous scoping review and people-centered care framework to identify potential roles, examining potential roles and expanding the roles’ framework, and presentation of the results in a tabular format for programmatic guidance is well appreciated.

**Response:** Thank you for your encouraging feedback. We appreciate your recognition of our methodological approach and the inclusion of both UAPs and NTEP-affiliated personnel, which we believe strengthens the relevance and programmatic value of our findings.

4. The results tables 2 and 3 identify the role as “Accompany suspected TB cases to a health facility”, whereas the associated text says presumptive. In line with use of respectful language, I suggest avoiding use of “suspect”. Please refer to the language guide: <https://www.stoptb.org/words-matter-language-guide>

**Response:** Thank you for this thoughtful and important suggestion. We fully agree that language plays a crucial role in promoting dignity and reducing stigma in tuberculosis care. In response, we have replaced all instances of “suspected TB cases” with “presumptive TB cases” throughout the manuscript, including in Tables 2 and 3, to ensure consistency with the associated text and align with the *Stop TB Partnership’s Words Matter* language guide. In addition, to further adhere to the guidance on respectful and people-centered language, we have also replaced “TB patients” with “people with TB,” “TB cases” with “people with TB,” and “TB suspect” with “person with presumptive TB.” These changes have been made consistently throughout the manuscript.

5. The focus is on UAPs. Although it is defined early-on, I disagree with the use of IP in lieu of UAP. The introduction mentions that only UAP subset of IP was involved and that should come across the entire paper and abstract. Using UAP instead of IP can be misleading by increasing the scope of interpretation to the different types of IP.

**Response:** Thank you for this important observation. We understand the concern that using the broader term “informal healthcare providers (IPs)” rather than “untrained allopathic practitioners (UAPs)” may imply a wider scope than intended. However, as discussed in our previous scoping review on this topic (Thapa et al., *PLOS ONE*, 2021; https://doi.org/10.1371/journal.pone.0256795), the literature on IPs is marked by considerable inconsistency in terminology, with terms such as IPs, UAPs, village doctors, and rural medical practitioners often used interchangeably. In that review, we used “informal healthcare providers” as an umbrella term and proposed a classification system in which UAPs form a distinct subgroup. To maintain consistency with the published literature and avoid introducing new terminology that may confuse readers, we have continued to use the term “informal healthcare providers” in this manuscript.

We have clearly defined in the introduction that our study focuses specifically on the UAP subgroup of IPs and discuss this limitation in the relevant section of the manuscript. In response to your suggestion, we have now added a clarification in the abstract stating that the study focuses on UAPs as follows:

*Considering such gaps, this study explores the perspectives of IPs (specifically untrained allopathic practitioners, UAPs) and NTEP-personnel regarding IPs' current and potential roles in TB care.*

**Please refer to line 29 on page 2 of the revised manuscript (clean version).**

6. On a related note, the authors decision to involve various levels of formal providers has improved the rigor of this work. However, formal providers involved were “three state-level officials, six District Health Office members, and two ASHA.” I would suggest authors to use another term instead of FP because typically FPs mean trained clinicians. The above set of people will be NTEP officials and community health workers. It is an overreach to call them FP, which should have included physician, other health workers, and potentially street-level NTEP staff. I note that authors do mention some additional details in limitation but that is insufficient.

**Response:** Thank you for this helpful and thoughtful suggestion. We agree that the term “formal providers (FPs)” is commonly interpreted to refer to trained clinicians, such as physicians, nurses, and other facility-based healthcare workers. In our study, however, the participants included a diverse group of individuals formally working within the National Tuberculosis Elimination Program (NTEP) structure at the state, district, and community levels, including program officials and Accredited Social Health Activists (ASHAs). To avoid confusion and more accurately reflect the roles of these participants, we have replaced the term “formal providers” with “NTEP personnel” throughout the manuscript. This terminology better captures the administrative and community-based nature of the roles represented in our sample.

We have also revised the Methods section to clearly define this group as individuals formally working within the NTEP structure.

*The second group of participants consisted of NTEP-personnel working in the NTEP of India at the state, district, and community levels, thereby representing various health system tiers. This group included personnel responsible for policy and program implementation at the state and district levels, as well as Accredited Social Health Activists (ASHAs) who deliver TB care services at the community level. Throughout this study, the term "NTEP-personnel"* *refers to individuals formally working within the NTEP structure, including administrative staff and community-based health workers such as ASHAs.*

**Please refer to line 169 on page 6 of the revised manuscript (clean version).**

In addition, we have updated the Limitations section to explicitly acknowledge that our study did not include formal healthcare providers such as physicians, whose perspectives may differ, particularly in relation to service delivery roles.

*Third, the study was limited to the views of NTEP-personnel up to the state level and did not include formal healthcare providers such as physicians, whose perspectives may differ, particularly with service delivery roles.*

**Please refer to line 585 on page 21 of the revised manuscript (clean version).**

7. As per table 3 on potential role, UAPs do not wish to accompany presumptive people to the facility. It would be useful to understand the underlying details in the accompanying text. Was this response universal or a one-off? Whom did it come from – UAP or a certain FP cadre? What were their reasons?

**Response:** Thank you for this thoughtful comment. We have added a clarifying sentence in the relevant section of the findings to elaborate on the views regarding this role. Specifically, we have highlighted that this perspective was expressed only by IPs and was attributed to time constraints and disruption to their routine work. Importantly, NTEP-affiliated personnel did not discuss this role during the interviews, either as a current or potential role. No additional information on this role emerged from our analysis.

**Please refer to line 359 on page 13 of the revised manuscript.**

8. As the introduction clearly explains, the policies for IP vary between states. It would be helpful to contextualize the NTEP’s approach. For instance, does NTEP have an approach towards engaging them? Are there any incentives – financial or other – for their engagement? Are they part of public-part mix or similar programs? Can they notify people with TB directly to the NTEP?

**Response:** Thank you for this valuable comment. We have revised the relevant section of the Introduction to provide greater context regarding the NTEP approach to engaging IPs. Specifically, we now clarify that the National Strategic Plan (NSP) recommends engaging all types of providers, including informal ones, under the public–private mix (PPM) framework. While the formal private sector has seen significant engagement, formal inclusion of IPs in most states remains limited, with West Bengal (WB) being one of the few exceptions. We have expanded this section to include details from our previous work, which documented gaps in the implementation of IP engagement in WB, such as reliance on paper-based referral systems and irregular disbursement of referral incentives. We also highlight the contrast between the clear policies for engaging formal private providers and the relative lack of policy guidance for involving IPs in TB care.

**Please refer to line 87 on page 4 of the revised manuscript (clean version).**

9. “Therefore, the use of the term IPs in this review only represents this group of providers”. (Lines 181-182). Did you mean “Therefore, the use of the term IPs in this review represents only this group of providers.”?

**Response:** Thank you for this helpful suggestion. We have revised the sentence for clarity. The updated sentence now reads: “Thus, the use of the term ‘IPs’ in this paper henceforth refers only to this subgroup of providers (UAPs).”

**Please refer to line 117 on page 5 of the revised manuscript.**
