# Supplementary material for: Exploring current and potential roles of informal healthcare providers in tuberculosis care in West Bengal, India: A qualitative content analysis
Source: PLOS Glob Public Health. 2025 Nov 5;5(11):e0004085. doi: 10.1371/journal.pgph.0004085 (PMC12588489; doi:10.1371/journal.pgph.0004085)
Supplement: Attachment — Submitted filename: Rebuttal letter.docx [file pgph.0004085.s007.docx]

22 August 2025

The Editor,

PLOS Global Public Health,

Dear Editor,

**Re: Exploring current and potential roles of informal healthcare providers in tuberculosis care in West Bengal, India: a qualitative content analysis**

We thank you and the external reviewers for the valuable comments that have allowed us to strengthen our manuscript. We have carefully reviewed the manuscript and provide a detailed response to the reviewers' comments below. We have also provided a tracked change version of the manuscript, highlighting the changes made to the originally submitted version.

**Reviewer 1:**

**Thanks for considering the comments. The current version reads very well after the exhaustive and careful revision. I have no further comments.**

**Response:** Thank you for your valuable comments on our previous submission, which have helped us significantly to improve the quality of our manuscript.

**Reviewer 2:**

**1. Specify the type of qualitative design utilised in the study. Line 178 suggests it was exploratory in nature.**

**Response:** We appreciate your observation. In response, we have revised the description of our study design to explicitly state that this was an exploratory qualitative design, appropriate for investigating under-researched or complex topics. The revised paragraph now reads as follows:

*This study employed an exploratory qualitative design, appropriate for examining under-researched or complex phenomena from participants’ perspectives. The analysis was guided by the principles of content analysis [29].*

**2. Although the previous study was cited and purposive sampling was mentioned, briefly describe how participants were recruited into the main study. How were prospective participants identified, approached and recruited? How many participants refused to participate in the study? If any, what rationale did those who refused to participate in the qualitative study give?**

**Response:** Thank you for this helpful comment. We have now clarified and described how participants for the qualitative study were recruited in the Methods (line 164). We have added the following revised paragraph to the manuscript:

*This qualitative study was nested within a two-phase research project conducted in the Birbhum district. In Phase 1, a knowledge survey was carried out with a sampling frame of 589 IPs, developed in collaboration with the LF and the local IPs association [1]. These IPs were located across all 19 administrative blocks of Birbhum, delivering care in community settings through private clinics and home-based visits. In Phase 2, 203 IPs from this initial frame participated in a second survey on TB care practices. Eligibility for the Phase 2 survey required that providers had managed at least one confirmed TB case in the preceding six months [2]. This criterion aligned with the objectives of this qualitative study, which aimed to explore the current and potential roles of IPs in TB care based on their recent experience.*

*During Phase 2, verbal consent for future re-contact was obtained from participants, allowing researchers to approach them for participation in subsequent research. For this qualitative study, the inclusion criteria were: (a) participation in the Phase 2 survey and (b) willingness to take part in a follow-up interview. From the pool of 203 eligible IPs, 30 were purposively selected by two researchers (PT and KB) to ensure variation in age, education, and years of experience. Twenty-three agreed to participate, while seven declined due to time constraints, as they were busy with service delivery related to the COVID-19 pandemic.*

**3. Specify the location within the district from which participants were sampled. Eg. In health facilities, within communities?**

**Response:** We thank you for this important suggestion. We have clarified in the revised manuscript in the Methods (line 166) that the primary sampling frame (Phase 1) included IPs working across all 19 administrative blocks of Birbhum districts, delivering healthcare services within the community through private clinics or via home-based visits. We have added the following information to the manuscript.

*In Phase 1, a knowledge survey was carried out with a sampling frame of 589 IPs, developed in collaboration with the LF and the local IPs association [1]. These IPs were located across all 19 administrative blocks of Birbhum, delivering care in community settings through private clinics and home-based visits.*

**4. Although participant availability is mentioned as a requirement for participation, specify the inclusion and exclusion criteria for both populations/groups of participants, ie, IPs and NTEP personnel.**

**Response:** Thank you for this helpful comment. We have now clearly outlined the inclusion criteria for both informal healthcare providers (IPs) and NTEP-affiliated personnel in the revised manuscript in the Methods (lines 174 and 180).

For IPs, we added the following:

*Inclusion criteria for IPs in the qualitative study were: (a) participation in the Phase 2 survey, and (c) willingness to participate in a follow-up interview.*

For NTEP-affiliated personnel, we revised the relevant paragraph as follows:

*Inclusion criteria for the second group of participants were: (a) NTEP-affiliated personnel working in the NTEP of India at the state, district, and community levels and (b) willingness to participate in an interview.*

5. **Indicate the number of interviews conducted via phone/face-to-face.**

**Response:** We appreciate the reviewer’s request for clarification. We have added the following information in the method section (line 212).

*All interviews with IPs were conducted remotely by phone. For NTEP-affiliated personnel, four interviews were conducted in person, while the remaining seven were carried out via telephone.*

6**. Describe how trustworthiness was achieved in this qualitative study.**

**Response:** We thank the reviewer for this important comment. In the revised manuscript, we have elaborated on how trustworthiness was ensured, using Lincoln and Guba’s (1989) four criteria: credibility (pilot testing, in-depth interview tool and method, diverse participants), dependability (detailed methodology including coding process and methodology), confirmability (two participant groups, audit trail, shared coding), and transferability (detailed description of participants, context and research process) in methods, line 256.

**Text added to the manuscript:**

We used Lincoln and Guba’s (1989) four criteria – credibility, dependability, confirmability, and transferability to establish trustworthiness in this study, an accepted approach in the content analysis method [3, 4]. To ensure credibility, we pilot-tested the study tool, used an interview guide to provide broad and consistent coverage of topics, and conducted in-depth interviews with providers of different ages, genders, and educational backgrounds to capture variation in perspectives. To enhance dependability, we provide a transparent account of the study design, data collection, and analysis, and the coding framework was iteratively discussed and refined among researchers. To strengthen confirmability, we included two groups of providers (IPs and NTEP-affiliated personnel) to enable triangulation of perspectives, maintained an audit trail documenting coding decisions and revisions to the coding framework, and shared codes with the RA involved in data collection to ensure transparency. For transferability, we provided a detailed description of the study participants, the context, and the research process so that readers can assess the applicability of the findings to other similar settings.

7. **Reporting descriptive statistics (such as median age, percentage being majority, etc) may be problematic in a qualitative study, as this. Consider reporting the participants’ characteristics broadly and provide a table of key characteristics such as age, sex, education and work experience.**

**Response:** We thank the reviewer for this valuable observation. While we understand the caution around using quantitative descriptors in qualitative research, we believe that limited use of descriptive statistics can enhance clarity regarding the participant sample. In response to the suggestion, we have now included a summary table (Table 1, presented below) that presents key participant characteristics, including age, sex, education, and work experience.

**Table 1: Characteristics of Study Participants**

| **Characteristic** | **NTEP-Affiliated Personnel (n = 11)** | **Informal Healthcare Providers (n = 23)** |
| --- | --- | --- |
| Sex | Male: 7 (64%), Female: 4 (36%) | Male: 20 (87%), Female: 3 (13%) |
| Age (years), median (range) | 45 (30–58) | 43 (27–65) |
| Education | Secondary level: 2 (18%), Higher education: 9 (82%) | Secondary or below: 4 (17%), Post-secondary: 11 (48%), Higher education: 8 (35%) |
| Level of the health system | State level: 3 (27%), District level: 6 (55%), Community level: 2 (18%) | Not applicable |
| Work experience (years), median (range) | 14 (2–30) | 15 (3–40) |

8**. Provide a table which presents the main study findings (headings: 1, 1.1, 1.1.1) to orient readers.**

**Response:** We appreciate the reviewer’s helpful suggestion. To better orient readers to the main findings, we have added a new table (Table 2, included below) that summarises the overall major study findings (categories and subcategories of IPs' roles in TB care), as expressed by both IPs and NTEP-affiliated personnel. This table consolidates the current roles, potential roles, and roles deemed unsuitable for IPs, as determined through content analysis.

**Table 2: Summary of the overall study findings (main categories and subcategories of IPs' current and potential roles in TB care)**

| **S. N** | **Major categories of roles** | **Role’s subcategories, as expressed by IPs** | **Role’s subcategories as expressed by NTEP-affiliated personnel** |
| --- | --- | --- | --- |
| 1 | Current roles in TB care | 1. Passive case findings and referral, | 1. Passive case finding and referral |
|  |  | 2. Accompanying presumptive people with TB to a health facility | 2. Treatment supporter |
|  |  | 3. Collection/transportation of sputum samples |  |
|  |  | 4. Treatment supporter |  |
| 2 | Potential roles in TB care | 1. Health promotion and education | 1. Health promotion and education |
|  |  | 2. Active case finding | 2. Passive case finding and referral |
|  |  | 3. Passive case finding and referral | 3. Collection/transportation of sputum samples |
|  |  | 4. Collection/transportation of sputum samples | 4. Contact tracing |
|  |  | 5. Treatment supporter | 5. Treatment supporter |
|  |  | 6. Counselling – During TB treatment | 6. Counselling – During TB treatment |
| 3 | Roles not suitable for IPs |  | 1. Clinical evaluation of TB |
|  |  |  | 2. Treatment initiation |

9. **Table 2 is unclear. The representation of a related scoping review's findings in this paper, together with the results of this qualitative paper, is challenging, especially for qualitative research fundamentalists or purists. It may be useful to discuss how the results of previous papers relate to the findings in the discussion section.**

**Response:** We thank the reviewer for this very helpful comment. We have revised Table 2 (now Table 3 in the manuscript) to include only the findings from the current qualitative study. However, as per your suggestion, we have retained a brief discussion of the previous scoping review findings in the discussion section, where relevant.

**10. “Post-Sec” should probably be explained when used the first time or in the methods.**

**Response:** We thank the reviewer for pointing this out. We have revised the text to replace “Post-Sec” with the full term “post-secondary education” when first mentioned in the manuscript to ensure clarity for readers.

**11. It may be helpful to attribute quotes to participants with ID numbers, eg, IP 1, IP2, or IP23.**

**Response:** Thank you for this helpful suggestion. We agree that including participant ID numbers can offer an additional layer of clarity in qualitative reporting. In our case, we chose to provide contextual descriptors (participant type, age, sex, and education) alongside each quote, which offered sufficient context for interpretation. Revisiting the transcripts to assign numerical IDs at this stage would require considerable time and may not substantially enhance the readers' understanding beyond what is already presented. We hope our current approach remains acceptable.

**References cited in the revised manuscript:**

1.	Thapa P, Jayasuriya R, Hall JJ, Mukherjee PS, Beek K, Briggs N, et al. Are informal healthcare providers knowledgeable in tuberculosis care? A cross-sectional survey using vignettes in West Bengal, India. International Health. 2022;15(4):389-96.

2.	Thapa P, Hall JJ, Jayasuriya R, Mukherjee PS, Beek K, Das DK, et al. What are the tuberculosis care practices of informal healthcare providers? A cross-sectional study from Eastern India. Health Policy and Planning. 2022;37(9):1158-66.

3.	Guba EG, Lincoln YS. Fourth generation evaluation: Sage Publications 1989.

4.	Elo S, Kääriäinen M, Kanste O, Pölkki T, Utriainen K, Kyngäs H. Qualitative content analysis: A focus on trustworthiness. SAGE open. 2014;4(1):2158244014522633.
